# Narrative review of EHDI in South Africa

**DOI:** 10.4102/sajcd.v62i1.126

**Published:** 2015-11-27

**Authors:** Selvarani Moodley, Claudine Storbeck

**Affiliations:** 1 Centre for Deaf Studies, University of the Witwatersrand, South Africa

## Abstract

**Background:**

With 17 babies born with hearing loss every day in South Africa, there is a pressing need for systematic Early Hearing Detection and Intervention (EHDI) services. Progress is being made in offering newborn hearing screening and studies have been conducted to document these processes within South Africa. However, due to the lack of a national and holistic overview of EHDI services to date, an accurate picture of the current status of EHDI within the South African context is required.

**Objective:**

To document and profile what has been published within the field of EHDI in South Africa over the last two decades (Jan 1995–Sept 2014) in order to gain a comprehensive overview of the current status and practice of screening and diagnosis in the field of paediatric hearing loss.

**Method:**

A narrative review of peer-reviewed articles related to EHDI in South Africa was conducted by searching the EBSCOHOST, SCOPUS and JSTOR databases for the period January 1995 to September 2014.

**Results:**

Results indicate that over the last two decades research and publications in the field of EHDI have increased considerably. These publications have revealed extensive knowledge related to paediatric hearing screening and intervention services in South Africa; however, this knowledge seems to be limited primarily to the provinces of Gauteng and the Western Cape. Furthermore, studies pertaining to diagnosis have revealed that, although much has been written on the scientific aspects on tools for diagnosis of hearing loss, there is a lack of comprehensive information on diagnostic protocols and procedures.

**Conclusion:**

Despite the clear progress being made in South Africa in the field of early hearing detection and intervention, there is a need for comprehensive studies on protocols and procedures in diagnosing paediatric hearing loss. Finally, the narrative review revealed a clear need to ensure that development and growth in the field of EHDI is a national priority and extends beyond the two provinces currently showing growth.

## Introduction

South Africa is estimated to have 16–17 babies with hearing loss born daily (Swanepoel, 2008) and despite having no legislation in place, awareness of the importance of early detection of hearing loss, followed by appropriate intervention, is growing. Early Hearing Detection and Intervention (EHDI) describes the pathway from infant screening for hearing loss to the subsequent process of diagnosis and intervention.

In response to the international drive for effective and early EHDI programmes, the Health Professions Council of South Africa (HPCSA) published a position statement in 2007. This statement describes internationally benchmarked indicators of screening at birth, amplification by three months and enrolment in early intervention by six months of age (as published in Joint Committee on Infant Hearing [JCIH], 2007) and then provides the South African benchmarks of screening by four months, appropriate amplification by six months and enrolment in an early intervention programme by no later than eight months of age (Health Professions Council of South Africa [HPCSA], 2007).

Universal newborn hearing screening and the subsequent early diagnosis of infant hearing loss is a new practice in South Africa when compared to international progress. In the USA as early as 1965, health, education and welfare departments recommended universal evaluation of hearing loss on a national level. Hearing screening has since become universal in the USA (every newborn is offered the option of having a hearing screening), with only 5% of newborns not having their hearing screened (Eiserman et al., 2008). Although there is a system in place to ensure newborn hearing screening in the USA, the system is complex. Screening occurs at the birth hospital, rescreening at a different outpatient facility, referral to an otorhinolaryngologist for amplification and referral to early intervention services (Russ, Hanna, DesGeorges & Forsman, 2010). Quality management, which includes record keeping and reporting to ensure a seamless transition from screening to diagnosis to intervention, is ensured through a good data management system (Finitzo & Grosse, 2003).

In the United Kingdom (UK), hearing screening forms part of their general screening regimen and is preceded by screening procedures for medical disorders such as haemoglobin disorders, metabolic disorders and neurogenetic disorders. By 2004, as part of the Universal Newborn Hearing Screening Programme in England, between 90% and 94% of mothers had been offered hearing screening for their newborn (Bamford, Uus & Davis, 2005). Prior to implementation of universal hearing screening in the UK, studies were conducted on the likely impact of screening on paediatric audiology services. The readiness of paediatric audiology services to deal with referrals from newborn hearing screening and the proficiency of audiologists in the accurate diagnosis of and intervention for paediatric hearing loss were determined (Uus, Bamford, Young & McCracken, 2005). These studies informed the feasibility of the screening programmes as well as the training needs of audiologists involved in paediatric audiology screening and diagnosis. It also informed the development of guidelines for screening and diagnosis.

The implementation of effective hearing screening programmes as outlined in developed countries requires numerous resources and financial investment A number of conceptual and research articles have recognised the impracticalities inherent in attempting to implement developed world models of hearing screening in a developing country (Swanepoel, Delport & Swart, 2004; Swanepoel, Hugo & Louw, 2005a), and recommendations highlighted the need for further research in the areas of using alternative screening forums (Swanepoel, Hugo & Louw, 2005b, 2006) for effective implementation of Universal Newborn Hearing Screening (UNHS) in South Africa.

Although numerous screening programmes have been implemented in hospitals and local South African communities, research to report on the outcomes are isolated and broader studies describing a collated overview of the general knowledge base and status of EHDI in South Africa are missing. Furthermore, areas in which there are gaps in knowledge have not been systematically presented nor has there been an outlining of strengths and weaknesses of EHDI systems and identification of areas where additional research is necessary.

A review of published studies on EHDI in South Africa was therefore needed to gain a comprehensive understanding of the current practice and status of newborn screening and diagnosis of paediatric hearing loss.

## Research methodology

### Research design

This study, whilst sharing much in common with the systematic review in terms of an academic database search with clear parameters (Liberati et al., 2009), is descriptive rather than evaluative and therefore a narrative review. The overall aim of a narrative review is to ‘tell the story’ and thus provide an overall description of the current knowledge base and national activity within the field of EHDI as evidenced in the published research.

In telling the story about the publications, the aims of this narrative review are:

1. To detail the number of peer-reviewed papers on EHDI in South Africa from January 1995 to September 2014 and to classify the articles according to type of publication: a conceptual or research article or a letter to the editor.
2. To describe the main and secondary focus of each paper in order to get an overview of the work that has been done in the field within this time-frame.
3. To outline the chronological progress within the field of early hearing detection and intervention over the period of twenty years, January 1995 to September 2014.

### Data collection

An electronic academic-database search of EBSCOHOST, SCOPUS and JSTOR databases was conducted using a combination of search terms related to the general EHDI topics of screening and diagnosis of paediatric hearing loss in South Africa. The two search parameters required that all the articles had to be peer-reviewed – thereby excluding theses and dissertations – and published between January 1995 and September 2014 (Table 1).

**TABLE 1:** Description of the search results identifying articles for the narrative review.

Procedural steps	Number of reports	Description
a) Database search results	759	3 databases (EBSCOHOST, JSTOR and SCOPUS), 9 search strategies
b) Database search results –duplicates omitted	676	83 duplicates from the 5 searches were omitted
c) Database results examined for scope of review	41	676 titles and keywords were examined for relevance to the review topic. Where there was uncertainty about relevance abstracts were reviewed. 635 articles were omitted – 634 not relevant to topic and 1 citation
d) Time frame for review identified as from 1995 to September 2014	35	6 articles were omitted due to being published before 1995
e) Identification of additional reports relevant to review	8	Reference lists of articles reviewed to identify additional relevant articles
f) Reports included in review	43	Articles forming part of the final systematic review

Of the 759 articles identified using the search terms across the three databases as noted above, 83 were identified as duplicates. The remaining 676 articles were examined for relevance and 635 were omitted as being irrelevant. An additional six were omitted as they were published before 1995. The 35 remaining articles were studied and reference lists were reviewed in order to identify any additional relevant articles, from which eight were identified. A final total of 43 peer-reviewed articles were included in the narrative review. Masters and PhD dissertations, as well as the HPCSA Position Statement were excluded from the review, as they are not peer-reviewed academic articles.

### Data analysis

Articles were classified according to: (1) type of publication, whether it was conceptual, editorial or a research paper; (2) year of publication in order to determine chronological progression of EHDI in South Africa; and (3) primary and secondary focus of the publication. If the majority of the article related to a specific area, it was classed as the primary focus. If some reference was made to a specific area, but was not the main topic, it was classed as the secondary focus.

#### Results

43 peer-reviewed articles were identified in the field of EHDI in South Africa for the period January 1995 to September 2014. These included 15 conceptual articles, 26 research articles and two letters to journal editors. In classifying the papers by their primary and secondary areas of focus, the following four categories arose: (1) screening, which includes the process and forum for identification of hearing loss; (2) diagnosis, which involves the comprehensive testing and classification of the hearing loss; (3) intervention, which refers to the fitting of amplification and referral to early intervention services and (4) data management, which includes reference to the follow-up and referral network. A summary of the 43 papers can be found in Appendix A.

The analysis revealed that almost 60% (*n* = 24) of the papers focused on screening as their primary focus and just under 30% (*n* = 11) focused on diagnosis, whilst the remaining six focused on intervention (*n* = 5) and data management respectively (*n* = 1). Two articles (Swanepoel, 2006; Swanepoel & Störbeck, 2008) could not be classified into one of the four areas, as one was an article describing the profession of audiology and the other was an overview article describing EHDI knowledge (Table 2).

**TABLE 2:** Area of focus for articles included in narrative review.

Primary focus	Secondary focus					
	Screening	Diagnosis	Intervention	Data management	No secondary focus	Total
Screening	-	-	6	2	16	24
Diagnosis	-	-	1	-	10	11
Intervention	-	-	-	-	5	5
Data management	1	-	-	-	-	1
Total	1	0	7	2	31	41

**FIGURE 1:**
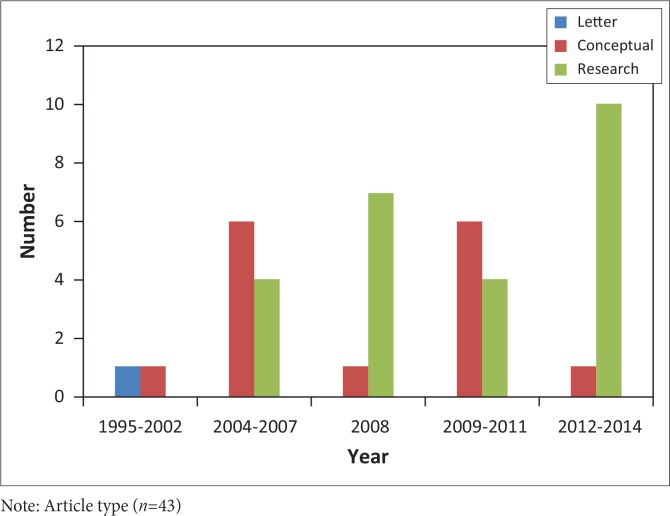
The chronological pattern. Note: Article type (*n*=43)

When considering the chronological pattern that emerged over the 20 years of publications, the research increased considerably over time (see Figure 1), with the first 8 years (1995 – 2002) showing no empirical research, the second eight-year period (2004 – 2011) showing a growth in conceptual papers and research papers with the largest growth in research occurring in the last 3 years (2012 – 2014).

The period of 1995–2002 showed minimal focus on the area of EHDI, with just two publications: one being a letter to the journal editor discussing the feasibility of introducing universal newborn hearing screening in South Africa (1995) and the other being a conceptual article discussing the Auditory Steady State Response (ASSR) as a tool for diagnosis of paediatric hearing loss.

More conceptual than research articles were published between 2004 and 2007. The focus of these articles was mainly on hearing screening forums and the importance of early identification and intervention for paediatric hearing loss. In 2008 a number of research articles dedicated to the proceedings of the first EHDI in Africa conference (held in 2007) was published in a special edition of the *International Journal of Audiology*. Between 2009 and 2010 the focus of the articles then shifted to a summary of the current status of EDHI services in South Africa and the introduction of a pilot screening programme implemented in one province of South Africa. The 2011–2014 period focused on alternative screening forums as well as retrospective reviews of audiology records to determine current practice.

## Discussion

The 43 articles included in the narrative review will be discussed chronologically, with reference to specific themes within each time period.

It is only in the new millennium (2000 onwards) that research in the field of EHDI has shown considerable growth in South Africa with studies in the specific areas of screening and early intervention. In 1993, the US National Institute of Health (NIH) Conference addressed the issue of late identification of hearing loss and made the recommendation of universal newborn hearing screening (NIH Consensus Statement, 1993). This led to a meeting at the Departments of Otolaryngology, Logopaedics and Neonatal Paediatrics at the University of Cape Town in South Africa. The conclusion of this meeting was that UNHS in South Africa was not feasible due to the lack of proof of effectiveness of hearing screening and the workload impracticality (Prescott, 1995).

Further research in this area then showed a decline until 2002, when general scientific research to determine the effectiveness of the ASSR in diagnosis of hearing loss in infants was conducted by South African authors (Swanepoel, Schmulian & Hugo, 2002).

The year 2004 also saw the importance of early identification and intervention for paediatric hearing loss being well recognised, with five conceptual articles (Swanepoel, 2006; Swanepoel, Delport & Swart, 2007; Swanepoel, Delport *et al*., 2004; Swanepoel, Louw & Hugo, 2007; Swanepoel et al., 2005a) and seven research articles relating to this topic published between 2004 and 2007. Research during this period focused mainly on the use of immunisation and maternal health clinics as forums for screening of hearing loss in infants (Olusanya & Okolo, 2006; Swanepoel, 2006; Swanepoel, Hugo & Louw, 2004; Swanepoel et al., 2005b, 2006; Swanepoel, Louw *et al.*, 2007), screening uptake at a private healthcare clinic (Swanepoel, Ebrahim, Joseph & Friedland, 2007) and one article focusing on the generic topic of the ASSR being a reliable measure of threshold estimation for children with severe to profound hearing loss (Swanepoel et al., 2002). Initial research in the field of EHDI from 2002 to 2007 focused mainly on an alternative screening forum that could be used in public health and the uptake of the offer for newborn hearing screening in private health. The use of screening methods, diagnostic data specific to South African practice and data management were gap areas in the research.

The areas of research and South African specific knowledge in the field of EHDI were highlighted when the first EHDI Africa conference was held in South Africa in 2007. A special supplement of the International Journal of Audiology (IJA) was dedicated to the publishing of proceedings from conference presentations. The introductory editorial article (Swanepoel & Störbeck, 2008) highlighted the increased concern and advocacy on the topic of paediatric hearing loss and EHDI on the African continent. Important findings from research in this special IJA supplement in terms of audiology included the lack of resources and personnel, as well as the need for resources and training in implementation of screening in the public health sector (Theunissen & Swanepoel, 2008), and in the fitting of paediatric amplification (Strauss & Van Dijk, 2008). The importance of the parents in the EHDI process and the role of the audiologist in providing on-going support and guidance (Swanepoel & Almec, 2008; Van der Spuy & Pottas, 2008), as well as the need to focus beyond screening and consider the importance of cultural, linguistic and accessibility factors in intervention (Störbeck & Pittman, 2008), was also addressed. Additional articles during this period focused on the scientific aspects of using the ASSR in the diagnosis of hearing loss (Swanepoel & Ebrahim, 2009; Swanepoel, Ebrahim, Friedland, Swanepoel & Pottas, 2008) and were not based on issues related specifically to the South African context. This period of research showed an expansion of earlier research in that there was a consideration of contextual factors related to staffing in the public health sector, the role of the audiologist in fitting of amplification as well as providing support. This included the importance of culture, linguistics and access to services in early intervention. However, there was still a lack of a focus on screening methods and effectiveness, South African specific diagnostic practice and data management.

The period of 2009–2010 produced a number of conceptual/review articles relating to the implementation of newborn hearing screening, early intervention and the importance of a follow-up referral network (Copley & Friderichs, 2010; Pillay, Moonsamy & Khoza-Shangase, 2010; Swanepoel, 2009; Swanepoel, Störbeck & Friedland, 2009). Research topics during this period focused on targeted screening and follow-up (Kanji, Khoza-Shangase & Ballot, 2010) and audiological management following meningitis (Khoza-Shangase & Rifkind, 2010). The need for screening and diagnostic protocols for children with a high risk of acquired hearing loss due to illnesses such as meningitis is unique to the African situation. Conclusions included the need for improved knowledge of hearing screening and early intervention among professionals as well as parents (Kanji et al., 2010; Khoza-Shangase, Barratt & Jonosky, 2010).

This highlights the importance of focused studies taking into account all aspects of the situation in Africa, including the professional knowledge of all specialists involved in the EHDI process, as well as the impact of ear infections and illnesses such as meningitis on acquired hearing loss. Data management and cross-disciplinary diagnostic data are clearly lacking in South African studies within the time period of the review.

Studies up until the 2009–2010 period focused mainly on the public healthcare sector, and thus provided little indication of EHDI services available to the 15% of the population that use private healthcare services. In 2011 and 2012 a survey of hearing screening practice in the private healthcare sector was conducted on a national level (Meyer & Swanepoel, 2011; Meyer, Swanepoel, Le Roux & Van der Linde, 2012). The inadequacy of hearing screening services in the private healthcare sector, the need to improve knowledge of hearing screening among professionals, as well as the need for a centralised data management and follow-up system was highlighted. In the public sector, a pilot screening programme using nurses as screening personnel in clinics in a single province of South Africa, the Western Cape, was implemented (Friderichs, Swanepoel & Hall, 2012). Conclusions from this programme were that follow-up is improved if there is a dedicated EHDI coordinator, and that screening coverage rates could be improved if screening was implemented at midwife obstetric units. The conceptual article outlining the many roles nurses can play in the EHDI process (Moodley & Störbeck, 2012) was contradicted by the pilot screening programme findings that for successful implementation of UNHS there needs to be dedicated EHDI personnel (Friderichs et al., 2012). This was further corroborated by a study in another province (Gauteng), where it was found that nurses do not adhere to documenting and recording screening protocols as proposed by the National Department of Health (Joubert & Casoojee, 2013). Whilst these studies acknowledge data management and follow-up as important factors, there is limited focus on the use of data management systems within the South African context. The use of midwife obstetric units (MOUs) as alternative screening forums is advancement on the studies earlier in the decade relating to the use of alternative screening forums. However, screening methods and the effectiveness related to this is an area that has still not been extensively studied. Until this period, South African specific diagnostic data and practice has not been researched.

More recent research activities (from 2012) have focused on retrospective and, less commonly, prospective review of audiology records. The prospective studies focused on using audiology records to determine the impact of risk factors for hearing loss in low birth-weight babies (Kanji & Khoza-Shangase, 2012) and a study of children with serous otitis media in one province (Tiedt et al., 2013). Findings from these studies correlated with previous findings of the late identification of hearing loss, audiology staff shortage, as well as that South African context specific risk factors should be developed if targeted screening is to be used for early identification of hearing loss. For targeted screening to be successful it is important for parents and healthcare professionals to recognise risk factors and that these children have access to hearing screening. Following findings that parent knowledge of hearing loss is poor, research conducted indicated that a contributing factor is the poor quality and readability of information pamphlets (Joubert & Githinji, 2014) and that parents refuse screening due to costs, as well as a lack of knowledge of the importance of hearing screening (Swanepoel, Scheepers & Le Roux, 2014). Whilst the focus of studies has moved on to investigate parent knowledge of hearing loss and screening and factors related to this, the main focus remains on screening of hearing loss. Even within the review of audiology records, there is a lack of comprehensive information on the process of diagnosing paediatric hearing loss in South Africa.

Studies following from screening to diagnosis, and the outlining of research related to diagnostic procedures and protocols, have only recently been conducted internationally (Larsen, Munoz, DesGeorges, Nelson & Kennedy, 2012; Munoz, Nelson, Goldgewicht & Odell, 2011). South African specific information related to diagnosis within the 2013/2014 period included two studies of retrospective audiology record analysis (Khoza-Shangase & Michal, 2014; Swanepoel, Johl & Pienaar, 2013). Whilst both these studies provide important initial information on diagnostic procedures, Swanepoel et al. (2013) focused on types of hearing loss related to risk factors and the diagnosis of auditory neuropathy spectrum disorder in a university clinic, whilst Khoza-Shangase and Michal (2014) focused on types of hearing loss, age of identification, amplification, intervention and a brief summary of diagnostic audiological evaluation in South African state hospitals. Neither of these studies provided an understanding of the process followed in diagnosis of type and level of paediatric hearing loss so as to allow for the progression to the next steps of EHDI, namely early intervention services and amplification. Previous South African studies related to diagnosis have focused on using the ABR and ASSR for the diagnosis of severe to profound hearing loss (Swanepoel & Ebrahim, 2009; Swanepoel, Hugo & Roode, 2004; Swanepoel et al., 2002), age of diagnosis of hearing loss in South Africa (Butler et al., 2013; Van der Spuy & Pottas, 2008), reports on diagnosis of hearing loss in state hospitals (Swanepoel, 2008) and protocols for audiological management following meningitis (Khoza-Shangase & Rifkind, 2010).

A study on audiology equipment and protocols used for paediatric assessment and hearing aid fitting highlighted the lack of both equipment as well as evidence-based guidelines for assessment in South Africa (Teixeira & Joubert, 2014). Developed countries have focused on putting policies and procedures in place to ensure early detection of childhood hearing loss and prompt initiation of early intervention services. Screening has become a standard of care, with studies on diagnosis being conducted to improve the diagnostic process. There is agreement on the need for standardisation and improvement of diagnostic services across developed countries.

The South African journey of research into EHDI services from 1995–2014 highlights the progress made in outlining the implementation of paediatric hearing screening services in a developing world context. The need for education of parents and professionals on the importance of early screening, the practical aspects of implementing screening programmes and the importance of South African specific risk factors being recognised was clearly highlighted. After the period of the review end date (September 2014) a further three articles were published in the field of EHDI in South Africa. One article focused on procedures and protocols for hearing screening at immunisation clinics in two South African provinces (Petrocchi-Bartal & Khoza-Shangase, 2014) whilst the other two focused on diagnosis and intervention for paediatric hearing loss (Le Roux, Swanepoel, Louw, Vinck & Tshifularo, 2015; Stroebel & Swanepoel, 2014). One focused on the parental experience of the diagnostic process for children with auditory neuropathy (Stroebel & Swanepoel, 2014) and the other focused on the risk profile and a summary of diagnostic procedures for children receiving cochlear implants (Le Roux et al., 2015).

Comprehensive research and evaluation of paediatric diagnostic audiology services and the procedures to be followed in the diagnosis of paediatric hearing loss in South Africa has emerged as a gap in the field. The use of tools such as the ASSR for diagnosis of paediatric hearing loss is an area of scientific knowledge for South Africa. However, comprehensive studies on diagnosis of paediatric hearing loss are lacking. The use of diagnostic tools and how they relate to the use of evidence-based guidelines for diagnosis of paediatric hearing loss, as well as the development of South African specific diagnostic guidelines (as was the case for recognition of the need for South African specific risk factors for screening), are areas for development and expansion.

An area of strength in EHDI research in South Africa is inclusive information on implementation of screening at different forums within the public and private healthcare sectors. The progress in development of South African specific risk factors with targeted screening as an option, as well as the need for the education of parents and professionals on the importance of hearing screening is clearly laid out. Whilst there is an acknowledgement of staff and equipment shortage for implementation of screening in the public health sector, there is not as much comprehensive information on the private health sector. An area that is also lacking is one that focuses on screening methods used and the effectiveness thereof. Related to this is what seems to be a lack of prioritisation of data management in the South African context, as few studies related to data management have been conducted

Limitations of this study include the fact that, as a narrative review, the study did not consider qualitative aspects of the research such as study sample or size, nor did it evaluate the research for possible bias or weakness in the individual studies. Additionally, the search, though exhaustive, did not include an independent researcher for verification.

## Conclusion

South Africa has taken many strides in growing the knowledge base of the field of EHDI, and has recognised and tried to overcome challenges in the implementation of EHDI services by researching and identifying alternative forums for screening, parent reasons for refusing screening and the availability of early intervention services. However, studies are mainly conducted in, and related to the context within, the Gauteng and Western Cape provinces of South Africa. Additionally, a large portion of the research is mainly focused on the screening process, with some studies into the usefulness of ASSR as a diagnostic tool and record reviews related to diagnostic procedures.

The research has shown that South Africa lacks a nationally agreed battery of tests and protocols for diagnosing hearing loss for infants and babies, and although a draft document on diagnostic guidelines for the paediatric population has been released for comment by the HPCSA, (Swanepoel, 2012), no process has yet been put into place to take it further. In addition, the current practice of audiologists in the diagnosis of paediatric hearing loss has not been fully explored or documented. Since accurate diagnosis is necessary for appropriate amplification and intervention and screening has been the primary focus to date, there is a need for studies focusing on the diagnostic process. The development of diagnostic practice guidelines appropriate for use in a developing country context will go a long way towards improving the EHDI process in Africa.

Studies looking at the development of universal screening, diagnosis and intervention across both the public and private healthcare systems will provide much needed information on all aspects of EHDI in a developing world context.

## Appendix

**Table:** Appendix 1: Database and search strategy details

Search strategy	Identifiers	Database	Number of articles
Articles related to early hearing detection and intervention in South Africa	Early hearing detection AND “South Africa”	Ebscohost Web-Academic Search Complete	4
		JSTOR	5
		SCOPUS	21
	Hearing loss AND ”South Africa”	Ebscohost Web-Academic Search Complete	15
		JSTOR	313
		SCOPUS	123
Articles focusing on screening for paediatric hearing loss in South Africa	hearing screening AND ”South Africa”	Ebscohost Web-Academic Search Complete	8
		JSTOR	9
		SCOPUS	57
Articles focusing on diagnosis of paediatric hearing loss in South Africa	“ hearing loss” AND “diagnosis” AND ”South Africa” in title	Ebscohost Web-Academic Search Complete	96
	“diagnosis of paediatric hearing loss” AND South Africa”	JSTOR	33
	Diagnosis of hearing loss” AND ”South Africa”	SCOPUS	75
	“Diagnosis of hearing loss”		
	“Diagnosis of deafness		

**Table:** Appendix 2: Summary of articles included in review

	Title	Year of Publication	Article Type	Article focus
1	Should routine screening of neonates for deafness be introduced in SA	1995	Letter to journal editor	Discussion of international introduction of UNHS and whether this should be introduced in SA
2	The effectiveness of the auditory steady state response in diagnosing hearing loss in infants	2002	Conceptual	A review of the Auditory Steady State Response as a procedure for the diagnosis of hearing loss in infants
3	Universal newborn hearing screening in South Africa: a First-World dream?	2004	Editorial	The importance of early hearing detection and intervention in both a first world and developing country context is outlined
4	Auditory steady state response for children with severe to profound hearing loss	2004	Research	The clinical usefulness of the Auditory Steady State Response in estimating behavioural thresholds for children with severe to profound sensorineural hearing loss
5	Implementing infant hearing screening at maternal and child health clinics: Context and interactional processes		Research	A qualitative description of the context and interactional processes observed at two maternal and child health clinics in a single province of South Africa
6	Infant Hearing Screening in the Developing World: rethinking First World Models	2005	Conceptual	The lack of feasibility of first world models of screening before hospital discharge is explained, and alternative screening forums and protocols proposed.
7	Audiology in South Africa	2006	Conceptual	The history and development of audiology as a profession in South Africa is presented
8	Infant hearing screening at immunization clinics in South Africa	2006	Research	510 infants enrolled at a clinic hearing g screening programme were analysed in terms of DPOAE coverage, high frequency tympanometry, and follow-up screening appointment attendance
9	Early hearing detection at immunization clinics in developing countries	2006	Conceptual	Description of the importance of early hearing detection and intervention as standard part of neonatal care in the developed world. Practical and culturally appropriate options for EHDI are being explored in developing countries.
10	Newborn hearing screening in a South African private health care hospital	2007	Research	A retrospective study of data from a screening programme at private hospital in Gauteng.
11	A novel service delivery model for infant hearing screening in developing countries	2007	Conceptual	A service delivery model for the implementation of infant hearing screening in developing countries is proposed.
12	Equal opportunities for children with hearing loss by means of early identification	2007	Editorial	Evidence for the importance of early hearing detection and intervention, nationally and internationally is presented
13	EHDI Africa: Advocating for infants with hearing loss in Africa	2008	Editorial	An introduction to articles related to conference proceedings from “Building Bridges in Africa”, the first EHDI Africa international conference held in South Africa in 2007.
14	Early hearing detection and intervention services in the public health sector in South Africa	2008	Research	The status of newborn infant hearing screening in the public sector (44 of 86 speech therapy and audiology departments). Diagnostic procedures used at 13 sites and data management at 12 sites is presented
15	Infant hearing loss in South Africa: age of intervention and parental needs for support	2008	Research	Questionnaire surveys and focus groups conducted with parents of children diagnosed with hearing loss
16	Early intervention in South Africa: moving beyond hearing screening	2008	Research	Data of 32 infants registered in a home-based early intervention programme is outlined
17	Maternal views on infant hearing loss and early intervention in a South African community	2008	Research	A survey of 100 mothers attending an immunization clinic in Gauteng, regarding their views and attitudes on infant hearing loss, was conducted.
18	Hearing instrument fitting of preschool children : Do we meet the prescription goals	2008	Research	Comparison of hearing instrument output values at 65 and 90dBSPL input signals fitted to preschool children to targets prescribed by DSLm across the frequency range.
19	Auditory steady-state responses to bone conduction stimuli in children with hearing loss	2008	Research	Investigation of bone conduction ASSR thresholds in children with normal hearing and various degrees of hearing loss
20	Auditory steady-state response and auditory brainstem response thresholds in children	2009	Research	Comparison of ASSR and ABR thresholds in 48 infants with varying degrees and type of hearing loss.
21	Identifying infant hearing loss - never too early, but often too late	2009	Editorial	The importance of prompt referral of children for hearing testing and the process of early identification is outlined
22	Early hearing detection and intervention in South Africa	2009	Conceptual	Review of the evidence and status of infant hearing loss and early hearing detection and intervention services in South Africa
23	Early detection of infant hearing loss in South Africa: editorial	2009	Editorial	A summary of the current status of EHDI in South Africa, together with evidence of the importance of developing infant hearing screening programmes on a wider scale
24	Paediatric meningitis and hearing loss in a developing country : Exploring the current protocols regarding audiological management following meningitis	2010	Research	Retrospective review of medical records of 47 children admitted with a diagnosis of meningitis to 2 academic hospitals in the Gauteng province.
25	Hearing screening follow up return rate in a very low birth weight project. A retrospective record review	2010	Research	Retrospective analysis of records of patients with low birth weight to determine the follow-up return rate in a hearing screening programme
26	An approach to hearing loss in children	2010	Conceptual/ Review	Difficulty on diagnosis of hearing loss in young children is outlined. The Ivan Toms Infant Hearing Screening Programme in Cape Town is presented as a pilot project for future UNHS programmes in South Africa
27	Protocols for early audiology intervention services: Views from early intervention practitioners in a developing country	2010	Research	Identification of the protocols and practices for early intervention in Gauteng government hospitals and how they compare to principles for effective early intervention.
28	Bridging the gap between early identification and intervention in the paediatric population with hearing impairment	2010	Conceptual	Description of the development of the Wits Hearing Aid Bank to bridge the gap between identification of hearing loss and actual intervention.
29	Newborn hearing screening in the private health care sector - a national survey	2011	Research	Hearing screening procedures implemented at and challenges in implementing screening in the private health care sector of South Africa
30	Clinical Status of the Auditory Steady-State Response in Infants	2011	Conceptual	The strengths and limitations of the ASSR for use with infants as well as the role of the ASSR in the paediatric test battery is presented.
31	Efficacy of a community based infant hearing screening program utilising existing clinic personnel in Western Cape South Africa	2012	Research	Evaluation of a UNHS programme with nurses trained as screening personnel at clinics in the Western Cape
32	Childhood hearing loss and risk profile in a South African population	2012	Research	Retrospective record review of 100 patient files to determine the hearing loss and associated risk profile of children diagnosed at a paediatric referral clinic
33	The occurrence of high risk factors for hearing loss in very low birth weight neonates: a retrospective exploratory study of targeted hearing screening	2012	Research	Determining the type and frequency of risk factor for hearing loss, as a backdrop for the argument for or against targeted hearing screening
34	Early detection of infant hearing loss in the private health care sector of South Africa	2012	Research	A national survey of EHDI services in the private healthcare sector of South Africa
35	The role of the neonatal nurse in early hearing detection and intervention in South Africa	2012	Conceptual	The multiple roles nurses play in the early identification and intervention of children with hearing loss is outlined.
36	Age of diagnosis for congenital hearing loss at Universitas Hospital, Bloemfontein	2013	Research	A retrospective, descriptive study to determine the age of diagnosis of hearing loss in the Free State province
37	Hearing screening record keeping practices at primary healthcare clinics in Gauteng	2013	Research	Review of hearing screening and record keeping practices at 20 primary healthcare clinics in Gauteng, south Africa
38	Paediatric chronic suppurative otitis media in the Free State province: Clinical and audiological features	2013	Research	A prospective study of the audiological, ontological and bacteriological findings of children (113 ears) with chronic serous otitis media at an academic hospital in the Free State province
39	Quality and readability of information pamphlets on hearing and paediatric hearing loss in the Gauteng Province, South Africa	2014	Research	The quality and readability of information pamphlets relating to hearing loss at hospitals in the Gauteng province were evaluated (n=21)
40	Why parents refuse newborn screening and default on follow up screening a South African perspective	2014	Research	A retrospective record review of 2 universal newborn hearing screening programmes to determine parent reasons for screen refusal and follow-up default
41	Why parents refuse newborn hearing screening in South Africa?	2014	Response to letter to editor	A response to a letter to the editor regarding the article “Why parents refuse newborn screening and default on follow up screening a South African perspective”
42	Early Intervention in Audiology: Exploring the Current Status from a Developing Country Context	2014	Research	Retrospective record review of the files of 70 patients between birth and 3 years from 3 state hospital audiology clinics in Gauteng
43	Availability of audiological equipment and protocols for paediatric assessment and hearing aid fitting in Gauteng, South Africa	2014	Research	A cross-sectional survey to determine the availability of audiological equipment and protocols for paediatric assessment and hearing aid fitting was conducted in the Gauteng province
